# Treatment of Post-Hypoxic Myoclonus using Pallidal Deep Brain Stimulation Placed Using Interventional MRI Methods

**DOI:** 10.5334/tohm.544

**Published:** 2020-10-13

**Authors:** Fay Gao, Jill L. Ostrem, Doris D. Wang

**Affiliations:** 1 Department of Neurology, Movement Disorders and Neuromodulation Center, Weill Institute of Neurosciences, University of California, San Francisco, San Francisco, CA, US; 2 Department of Neurological Surgery, University of California, San Francisco, San Francisco, CA, US

**Keywords:** myoclonus, GPi, iMRI, DBS, Lance-Adams syndrome

## Abstract

**Background:**

Post-hypoxic myoclonus (PHM) is characterized by generalized myoclonus after hypoxic brain injury. Myoclonus is often functionally impairing and refractory to medical therapies. Deep brain stimulation (DBS) has been used to treat myoclonus-dystonia, but few cases of PHM have been described.

**Case report:**

A 33-year-old woman developed severe, refractory generalized myoclonus after cardiopulmonary arrest from drowning. We performed MRI-guided asleep bilateral pallidal DBS placement, resulting in improvement in action myoclonus at one year.

**Discussion:**

Our case contributes to growing evidence for DBS for PHM. Interventional MRI guided DBS technique can be used for safe and accurate lead placement.

**Highlights:**

We report a case of a patient who developed post-hypoxic myoclonus after cardiopulmonary arrest from drowning, who later underwent deep brain stimulation to treat refractory myoclonus. This is the first case to describe asleep, interventional MRI-guided technique for implanting DBS leads in post-hypoxic myoclonus.

## Introduction

Post-hypoxic myoclonus (PHM) is characterized by generalized myoclonus with rest, action, and stimulus-provoked components, occurring after global hypoxic brain injury, in most cases due to cardiopulmonary arrest [1]. For patients who survive the initial hypoxic event, some develop chronic PHM, also known as Lance-Adams syndrome (LAS), days or weeks after regaining consciousness. In conjunction with other neurological symptoms from widespread cortical injury, including dysarthria, ataxia, postural lapses, and seizures, PHM can lead to debilitating functional impairments, with pharmacologic options limited to benzodiazepines and anti-epileptic medications [1,2,3]. However, up to 50% of patients may not respond to pharmacologic therapy [3]. While dysarthria and ataxia may improve with time, action myoclonus often persists for years despite optimal medical therapy [4].

Deep brain stimulation (DBS) is a well-established treatment for many movement disorders including essential tremor, Parkinson’s disease, and dystonia. In recent years, investigations into the utility of DBS to treat myoclonus-dystonia, a genetic disorder due to DYT11 mutation, have yielded promising results [5,6]. We identified four other reports that described use of DBS to improve chronic PHM, with three targeting the globus pallidus internus and one targeting the ventral intermediate nucleus of the thalamus [7,8,9,10], all using awake stereotactic technique with microelectrode recording.

We present the first reported case of a patient with PHM who underwent interventional MRI-guided (iMRI) implantation of bilateral pallidal DBS. Our patient had significant improvement in her action myoclonus after DBS, similar to the two other reported cases of patients with PHM treated with bilateral pallidal DBS [7,8]. We illustrate that asleep MR-guided DBS placement is safe and accurate and eliminates concerns associated with awake surgery. This report provides further evidence to the small but growing cases of DBS treating rare hyperkinetic movement disorders.

## Case Description

**Figure 1:**
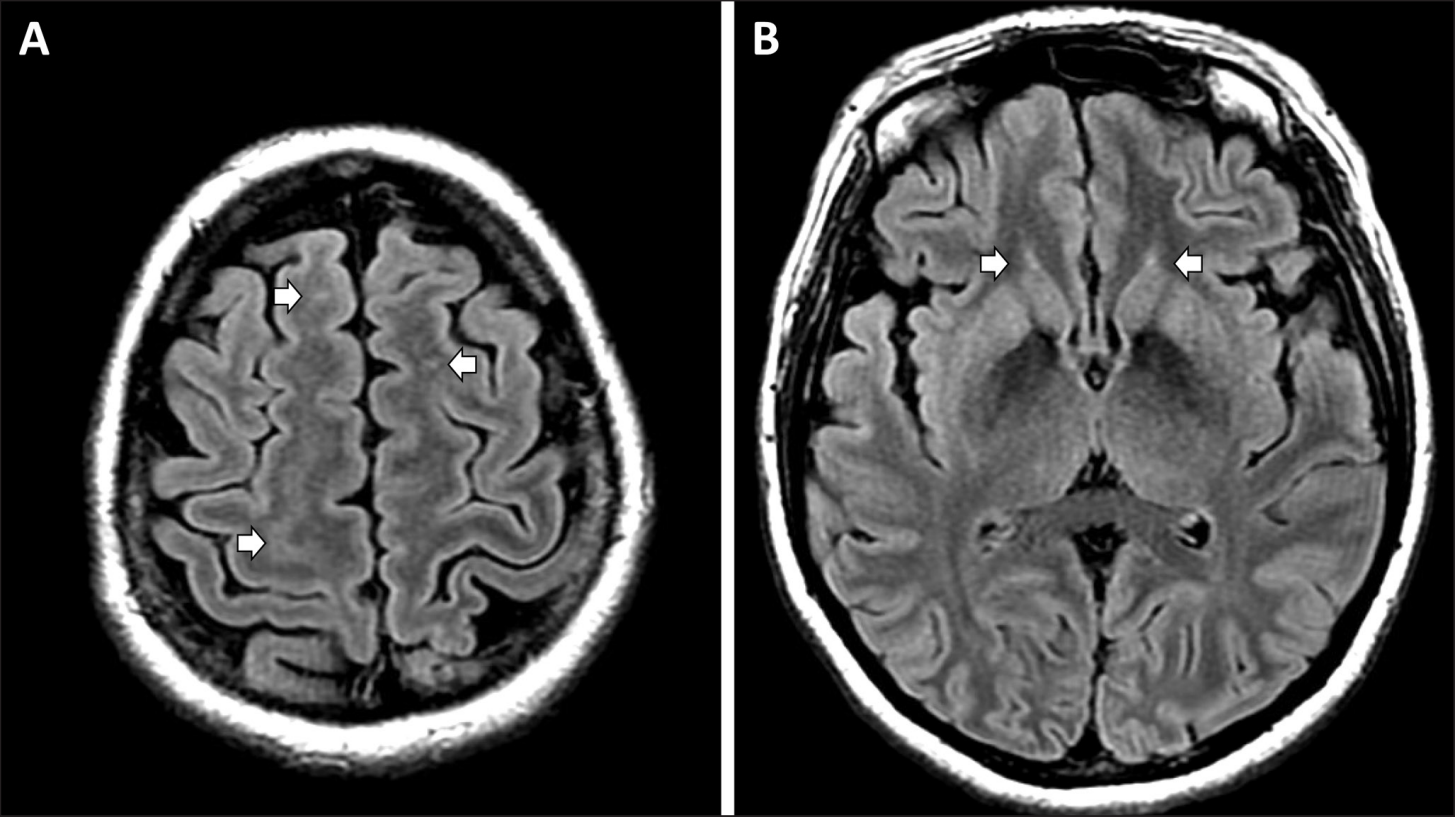
Preoperative MRI. **A.** Axial FLAIR-weighted preoperative MRI image showing diffuse cortical FLAIR signal hyperintensities as well as areas of FLAIR signal changes in the corona radiata (arrows). **B.** Axial FLAIR-weighted MRI image showing subtle FLAIR hyperintensities in the head of the bilateral caudate nuclei (arrows).

A 33-year-old woman was found in cardiopulmonary arrest after being submerged in a pool for 15 minutes. She was resuscitated and treated with hypothermia protocol. Complications included persistent respiratory failure requiring tracheostomy and gastrostomy tubes, as well as electrographic and clinical seizures characterized by generalized tonic-clonic movements and, later, behavioral arrests. She was treated with an antiepileptic drug (AED) regimen that included levetiracetam, clonazepam, phenobarbital, phenytoin, and lacosamide. After a month in a comatose state, she gradually recovered consciousness and regained language and sensorimotor functions. She was extubated and tracheostomy reversed. A brain MRI 1 month after initial injury showed subtle cortical and subcortical FLAIR signal hyperintensities in the corona radiata (Figure 1A) and the head of the caudate nuclei bilaterally (Figure 1B).

Over this time, she developed involuntary, large amplitude jerking of her limbs and trunk, without electrographic correlates, which were diagnosed as myoclonus. Laboratory workup revealed no clear metabolic disturbances, and EEG showed no electrographic correlates. Myoclonus remained during all waking hours that interfered with all activities. She could not sit or stand, and was dependent for feeding, cleaning, grooming, and toileting. Clonazepam and levetiracetam mitigated symptoms, but even with maximum titration, her myoclonus rendered her bedbound. She was discharged to an acute rehabilitation center but was frequently readmitted due to breakthrough seizures, urinary tract infections, and other complications from immobility. After five months of persistent myoclonus despite AED titration, she was referred to our center for consideration of DBS.

On examination, she was alert, oriented to time and location, spoke slowly and sparsely, and followed simple commands, but had impaired executive function, abstraction, and insight. Motor examination revealed mild symmetric weakness in the upper extremity extensor muscles and lower extremity flexor muscles, diffuse hyperreflexia, and normal tone. Coordination examination revealed dysmetria in proportion to weakness. She had minimal spontaneous jerks in the resting, supine position. On action, she had multifocal proximal and distal muscle jerks in her face, neck, trunk, and limbs, consistent with negative and positive myoclonus. Generalized jerks were also elicited by visual threat, clapping, and tactile stimulation in all extremities. She could not hold her limbs outstretched, use utensils, hold a cup, or sit upright (Video 1). There were no dystonic postures or movements. Given significant functional impairment and complications from myoclonus, our multidisciplinary movement disorder team offered DBS implantation as a palliative treatment for medication-refractory myoclonus. We chose the globus pallidus internus (GPi) as the target based on literature review [7,8,9,11].

**Video 1:** **Pre-DBS.** Demonstration of the patient’s resting and action myoclonus which impair her ability to perform functional tasks.

**Figure 2:**
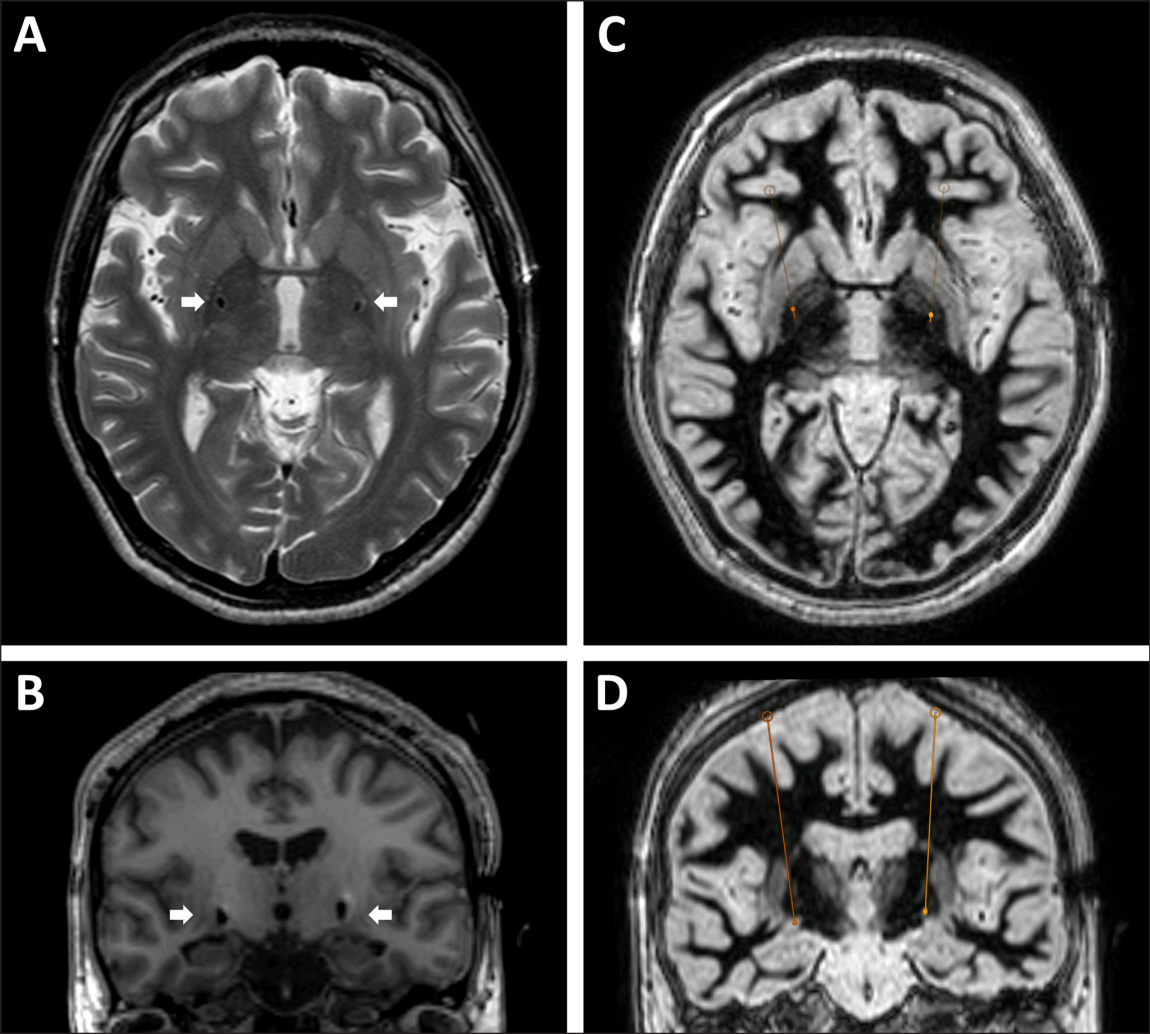
Pallidal DBS lead placement. **A.** Axial T2-weighted postoperative MRI image showing DBS lead artifacts (white arrows) at the AC-PC plane. **B.** Coronal T1-weighted posteropative MRI image showing DBS lead artifacts (white arrows) in the region of the posterior pallidum. C and D. Axial **(C)** and coronal **(D)** inversion recovery images showing borders of the globus pallidus externa and interna. DBS lead trajectories are indicated by orange lines. Intended left DBS target: 21 mm lateral, 2 mm anterior, 1 mm superior to MC with final DBS tip location extended 3.5 mm beyond the targeting plane to reach the base of the pallidum (above the optic tract). Intended right DBS target: 21.3 mm lateral, 2.3 mm anterior, 1 mm superior to MC with final DBS tip location extended 3.5 mm beyond the targeting plane. Left DBS lead location at targeting plane: 21.2 mm lateral, 1.9 mm anterior, 1 mm superior to MC (radial error of 0.3 mm). Right DBS lead location at targeting plane: 21.3 mm lateral, 2.3 mm anterior, 1 mm superior to MC (radial error of 0). MC=mid-commissural point (half point between anterior commissure to posterior commissure).

Seven months after the hypoxic event, she underwent asleep, iMRI-guided implantation of bilateral DBS electrodes in GPi (Medtronic 3389, Medtronic Inc., St. Paul, MN) (Figure 2). Targeting of the GPi based on iMRI-guided technique was performed as previously described [12,13]. This yielded radial errors of 0.3 mm in the anteromedial direction for the left side and 0 mm for the right side at the targeting plane (1 mm superior to the mid-commissural plane). One pass was performed for each side. Total operative time for lead implantation was 216 minutes. Bilateral chest Activa SC pulse generators (Medtronic Inc., St. Paul. MN) were later implanted. Her postoperative course was complicated by severe incisional pain and headache, which was treated aggressively with intravenous and oral medications.

Initial programming was performed three days postoperatively. Monopolar review suggested motor benefit from contacts 1 or 2 for each DBS lead. Initial settings were C+,1-, pulse width 60 microseconds, frequency 180 Hz, and amplitude 1.0 V, bilaterally. On higher amplitudes, she reported severe headache which, while not clearly related to stimulation side effect, led us to take a cautious approach to programming. The patient was transferred back to her community hospital and eventually discharged to a rehabilitation center. She returned to our clinic for further programming two months postoperatively. There was no indication of significant benefit, so stimulation was increased bilaterally to 2.0 V, then slowly increased further to 3.2 V over a course of a month. At the nine-month visit, only slight improvement in symptoms was noted. To provide more benefit, a new double monopolar configuration with C+,1-2-, pulse width 60 microseconds, frequency 180 Hz, and amplitude 3.2 V was created bilaterally. After this change, her family reported that her myoclonic movements were notably improved, and later recalled this time as a major positive change in her quality of life. One year after surgery, she could grasp utensils to feed herself and drink water from a cup with some spillage. She could hold a toothbrush and use a comb. She sat upright in a chair without assistance and could stand for a few minutes with significant bracing and support, but could not ambulate (Video 2). AEDs were continued after surgery.

**Video 2:** **12 Months Post-DBS.** The patient’s myoclonic movements are reduced, and ability to perform functional tasks are improved.

Objective assessment using the Unified Myoclonus Rating Scale (UMRS) [14] showed 35% improvement in action myoclonus (from 62 to 40), and 16% improvement in the functional testing component (from 19 to 16). Resting myoclonus was minimal pre-DBS but abolished post-surgery (2 to 0). To directly assess the effect of stimulation on her myoclonus, we compared her clinical examination on and off DBS in clinic. However, no clear change was observed up to one hour after DBS was turned off. This may have been due to an inadequate “washout” period following deactivation, as well as confounding effect from AEDs taken a few hours before her visit. Plans for future visits include cautiously raising stimulation and exploring other parameters.

## Discussion

Our case is the third report of a patient with PHM treated with bilateral GPi DBS resulting in improvement of resting and action myoclonus. One report demonstrated an 80% improvement in action myoclonus [7] and another showed a 38% improvement [8] (Table 1). All three reports demonstrated complete resolution of resting myoclonus. Additionally, there is another report of a patient who received unilateral GPi DBS following a putamen hemorrhage and cardiac arrest due to pulmonary embolism, whose unilateral action myoclonus improved by 71% and resting myoclonus improved by 75% after DBS [9]. In a subset of these cases, including ours, there are ataxic movements that traditional DBS targets do not treat and may unmask or worsen [7], limiting the degree of functional score improvement in the UMRS which does not take into account changes to ataxia. In all cases, pre- and post-operative UMRS was used to assess DBS efficacy. It is possible improvement may be confounded by natural recovery. This limitation must be better characterized by future studies using more rigorous assessment of DBS stimulation effect, comparing UMRS in the DBS on and off states with longer (1–2 day) washout periods. Despite these limitations, we felt that the time course and degree of motor improvement occurring after DBS surgery suggested that DBS stimulation improved our patient’s myoclonus. Although she remained functionally limited even with stimulation, she and her family reported that stimulation improved her quality of life. Altogether, we feel that consideration of DBS for treatment of medically-refractory PHM is reasonable in the right surgical candidate.

**Table 1:** Summary of published cases of post-hypoxic myoclonus treated with bilateral pallidal deep brain stimulation.

Case	Age	Sex	Mechanism of hypoxic injury	Time from injury to DBS	Target site and method	Stimulation parameters	DBS Efficacy		

							Preop UMRS	Postop UMRS (Length of follow up)	% improve-ment

Current case	33	F	Asphyxia due to drowning leading to CPA	5 months	Bilateral GPi Asleep, iMRI guided	L and R: Double monopolar C(+),1(–),2(–) Amp: 3.2 V Freq: 180 Hz PW: 60 µs	Action: 61 Resting: 2	Action: 40 Resting: 0 (1 yr)	Action: 35% Resting: 100%
Ramdhani et al	23	M	Asthma attack leading to CPA	3 years	Bilateral GPi Awake, stereotactic	R: Monopolar C(+),3(–) Amp: 2.8 V Freq: 130 Hz PW: 90 µs L: Triple monopolar C(+),1(–),2(–),3(–) Amp: 2.5 V Freq: 130 Hz PW: 60 µs	Action: 52 Resting: 75	Action: 32 Resting: 0 (6 mo)	Action: 38% Resting: 100%
Asahi et al	54	M	Respiratory distress leading to CPA	1 year	Bilateral GPi Awake, stereotactic	L and R: Bipolar 1(–),2(+) Amp: 2.0 V Freq: 125 Hz PW: 60 µs	Action: 25 Resting: 8	Action: 5 Resting: 0 (6 mo)	Action: 80% Resting: 100%

UMRS: Unified Myoclonus Rating Scale; CPA: cardiopulmonary arrest; Amp: amplitude, Freq: frequency; PW: pulse width; L: left; R: right.

Previously published case reports of DBS for PHM used the awake stereotactic technique involving microelectrode recording and intraoperative testing for placement [7,8,9,10]. Here, we report the first use of asleep iMR-guided method for lead placement in PHM. This technique offers several advantages compared to awake surgery. First, patients with PHM may have cognitive and functional impairments that limit participation during awake procedures. Second, global cerebral hypoxia may cause tissue injury that renders microelectrode mapping challenging and inaccurate. Finally, because of cortical tissue loss or subcortical structural damage from the initial injury, gliosis may deflect lead trajectories; therefore, the ability to directly visualize lead pathway with serial MRIs can lead to highly accurate placement. The iMRI technique for DBS lead implantation can expand the application of DBS to treating patients who otherwise cannot tolerate awake surgery.

We conclude that pallidal stimulation is a safe and well-tolerated treatment for PHM and that asleep iMRI guidance is an effective, accurate method for lead placement. Additional clinical trials with more structured protocol for patient selection, systematic programming guidelines, and objective, blinded, assessment of improvement are needed. As DBS improves with more technological and surgical advances, we anticipate that its application to treatment of rare movement disorders will continue to grow.
